# Cerebellar Pathology in an Inducible Mouse Model of Friedreich Ataxia

**DOI:** 10.3389/fnins.2022.819569

**Published:** 2022-03-24

**Authors:** Elizabeth Mercado-Ayón, Nathan Warren, Sarah Halawani, Layne N. Rodden, Lucie Ngaba, Yi Na Dong, Joshua C. Chang, Carlos Fonck, Fulvio Mavilio, David R. Lynch, Hong Lin

**Affiliations:** ^1^Department of Neurology, Perelman School of Medicine, University of Pennsylvania, Philadelphia, PA, United States; ^2^Department of Pediatrics and Neurology, The Children’s Hospital of Philadelphia, Philadelphia, PA, United States; ^3^Audentes Therapeutics, San Francisco, CA, United States; ^4^Department of Life Sciences, University of Modena and Reggio Emilia, Modena, Italy

**Keywords:** frataxin, cerebellum, glutamatergic and GABAergic synapses, gene therapy, mitochondria dynamics

## Abstract

Friedreich ataxia (FRDA) is an autosomal recessive neurodegenerative disorder caused by deficiency of the mitochondrial protein frataxin. Lack of frataxin causes neuronal loss in various areas of the CNS and PNS. In particular, cerebellar neuropathology in FRDA patients includes loss of large principal neurons and synaptic terminals in the dentate nucleus (DN), and previous studies have demonstrated early synaptic deficits in the Knockin-Knockout mouse model of FRDA. However, the exact correlation of frataxin deficiency with cerebellar neuropathology remains unclear. Here we report that doxycycline-induced frataxin knockdown in a mouse model of FRDA (FRDAkd) leads to synaptic cerebellar degeneration that can be partially reversed by AAV8-mediated frataxin restoration. Loss of cerebellar Purkinje neurons and large DN principal neurons are observed in the FRDAkd mouse cerebellum. Levels of the climbing fiber-specific glutamatergic synaptic marker VGLUT2 decline starting at 4 weeks after dox induction, whereas levels of the parallel fiber-specific synaptic marker VGLUT1 are reduced by 18-weeks. These findings suggest initial selective degeneration of climbing fiber synapses followed by loss of parallel fiber synapses. The GABAergic synaptic marker GAD65 progressively declined during dox induction in FRDAkd mice, while GAD67 levels remained unaltered, suggesting specific roles for frataxin in maintaining cerebellar synaptic integrity and function during adulthood. Expression of frataxin following AAV8-mediated gene transfer partially restored VGLUT1/2 levels. Taken together, our findings show that frataxin knockdown leads to cerebellar degeneration in the FRDAkd mouse model, suggesting that frataxin helps maintain cerebellar structure and function.

## Introduction

Friedreich ataxia (FRDA) is a life-shortening autosomal recessive neurodegenerative disorder caused by deficiency of the mitochondrial protein frataxin (Campuzano et al., 1996; Herman et al., 2006). This deficiency leads to a multisystemic phenotype that includes cardiomyopathy, muscle dysfunction, and in some cases diabetes (Ristow et al., 2000; Tsai and Barondeau, 2010); however, neurological deficits remain the ubiquitous feature of FRDA. Although FRDA pathology is marked by loss of large dorsal root ganglion (DRG) cells and axons in the dorsal columns, loss of the dorsal spinocerebellar tract, and corticospinal tract degeneration, the mechanisms behind the relationship between frataxin deficiency and neuronal death remains elusive (Lynch et al., 2012). The cerebellar neuropathology in FRDA patients includes loss of large principal neurons and synaptic terminals in the dentate nucleus (DN) as well as possibly Purkinje cell injury and remodeling (Kemp et al., 2016). This pattern of neurodegeneration leads to progressive ataxia, dysmetria, and dysarthria, which significantly affect the patient’s life quality.

Our previous studies in the Knockin-Knockout (KIKO) FRDA mouse model have demonstrated early cerebellar pathology, including abnormalities of mitochondrial biogenesis and synaptic deficits (Lin et al., 2017, 2018). As studying such features in early human disease is difficult, it is important to understand whether they also occur in other murine FRDA models. The tetracycline promoter-controlled inducible mouse model of frataxin deficiency (FRDAkd model) partially recapitulates the disease phenotype including DRG neuronal loss, cardiomyopathy, and ataxia (Chandran et al., 2017). Such mice exhibit premature mortality, age-dependent body weight loss, and severe motor coordination deficits. The FRDAkd mouse model allows a subacute, potentially titratable method of frataxin deficiency, and physical and biochemical changes are potentially reversible with removal of doxycycline (dox)- mediated induction. In the present work, we examined the neuropathological and neurochemical features of the cerebellum in this model of FRDA in the context of its global frataxin deficiency.

## Materials and Methods

### Materials

FRDAkd transgenic mice were originally obtained from Drs. Geschwind and Vijayedran at UCLA, then bred with C57BL/6J mice (The Jackson Laboratory Stock No: 000664) to generate equal proportions of wild-type and FRDAkd mice (Chandran et al., 2017). All animals were treated according to protocols approved by the Children’s Hospital of Philadelphia Institutional Animal Care and Use Committee (IACUC; protocol 16–250). To induce frataxin deficiency, cohorts of transgenic mice at 2–6 months old and matching controls were fed a Dox-compounded chow diet (200 PPM Doxycycline, Animal Specialties, and Provisions, LLC., Quakertown, PA, United States) or regular chow diet. Mice harboring the shRNA transgene (Tet-O model) are given the acronym TG while WT refers to Wild-Type mice (Table 1). This is followed by (+), (−), or (±), reflecting Dox induction, withdrawal, and rescue/reversal, respectively. FRDAkd mice (TG +) were euthanized, and tissues were harvested after 4, 8, 9, 12, and 18 weeks of Dox administration. To attempt to reverse frataxin deficiency, mice were withdrawn from Dox food after 9 weeks of induction. Weight was monitored on a weekly basis upon induction of the frataxin shRNA. All mice were genotyped at weaning by a commercial vendor (Transnetyx, Cordova, TN, United States).

**TABLE 1:** Nomenclature and mice description.

Nomenclature	Mice description
Tg +	Transgenic [harboring the FXN shRNA transgene (Tet-O model)] treated with doxycycline
Tg-	Transgenic [harboring the FXN shRNA transgene (Tet-O model)] treated with no doxycycline
Tg ±	Transgenic [harboring the FXN shRNA transgene (Tet-O model)] mice that were treated with doxycycline food 9 weeks and 9 weeks without doxycycline (rescue)
WT +	Wild-type treated with doxycycline
WT-	Wild-type treated without doxycycline
WT ±	Wild-type treated with doxycycline for 9 weeks and 9 weeks without doxycycline (rescue)
PGK Half	Transgenic [harboring the FXN shRNA transgene (Tet-O model)] treated with doxycycline and at week 10 received one dose of AAV8-PGK-mFXN vector (0.5 E + 14 vg/kg) intravenous injection
PGK Low	Transgenic [harboring the FXN shRNA transgene (Tet-O model)] treated with doxycycline and at week 10 received one dose of AAV8-PGK-mFXN vector (1.00E + 14 vg/kg) intravenous injection
PGK High	Transgenic [harboring the FXN shRNA transgene (Tet-O model)] treated with doxycycline and at week 10 received one dose of AAV8-PGK-mFXN vector (3.00E + 14 vg/kg) intravenous injection
Placebo	Transgenic [harboring the FXN shRNA transgene (Tet-O model)] treated with doxycycline and at week 10 received one dose of vehicle (Lactated Ringer’s Solution w/10% Poloxamer: 1 μl/g) intravenous injection

Antibodies used included anti-GAD65/67 [Millipore, AB1511, 1:1,000 for western blotting (WB)], anti-VGLUT1 (Synaptic Systems, 135302, 1:2,000 for WB), anti-VGLUT2 (Synaptic Systems, 135402, 1:1,000 for WB), anti-frataxin (Abcam, ab175402, 1:250 for IHC and 1:1,000 for WB), anti-ATP5A (MitoSciences, MS507, 1:500 for IHC), anti-GAPDH (Novus Biologicals, 1D4, NB300-221, 1:1,000 for WB) and anti-actin [Abcam, ACTN5(04), ab3280, 1:5,000 for WB], anti-pDrp1 [(ser-616), 1:1,000 WB and 1:250 IHC, CST 3455S], Drp1 (1:1,000 Abcam 184247). Secondary antibodies for IHC included Alexa fluor 488 (Invitrogen, A11029, 1:250) or 594 (Invitrogen, A11036, 1:250) and for WB mouse or rabbit antibody (1:2,000 Cell Signaling Technology).

#### Vector and Viral Constructs

The adeno-associated viral vector AAV8-PGK-m*FXN* encoded the mouse frataxin cDNA, codon-optimized to avoid the knockdown by the endogenous shRNA from the FRDAkd mouse, driven by the human phosphoglycerate kinase 1 (PGK) promoter. The AAV vector was produced in HEK293 cells by two-plasmid transient transfection and purified by column chromatography. All vectors were manufactured by Audentes Therapeutics, and the titer and endotoxin levels were tested. Two lots were produced for each test article.

#### Gene Therapy-Treated Animals

Recombinant AAV8 vectors (AAV8-PGK-mFXN) were obtained from Audentes Therapeutics, AAV8-PGK-m*FXN*. At 10 weeks post dox treatment, FRDAkd mice (*n* = 75) were acclimated to the experimental room for 15 min or until tail vein dilation. The AAV8-PGK-m*FXN* vector was delivered to the FRDAkd mice at the indicated dose (Half 0.5 E + 14 vg/kg, Low IV 1.00E + 14 vg/kg, High 3.00E + 14 vg/kg) or vehicle (Lactated Ringer’s Solution w/10% Poloxamer—Injected: 1 μl/g) by intravenous (IV) tail vein injection.

### Tissue Preparation and Harvest

In initial experiments, 80 animals [WT, *n* = 45 (females and males), TG, *n* = 35 (females and males)] were sacrificed for tissue analysis. Animals at 2–6 months old were sacrificed after 4, 8–12, and 18 weeks of Dox induction. The cerebellum was dissected and rinsed in PBS before being transferred to 2 mL RNase-free tubes and set within dry ice. The collected tissue was stored at −80°C immediately. Tissue was prepared as previously described (Lin et al., 2005). Each respective tissue was homogenized in 20 ml lysis buffer per 1 g/weight and lysed for 1 h at 4°C. Lysis buffer containing 150 mM NaCl, 1 mM EDTA, 100 mM Tris–HCl, 1% Triton X-100, and 1% sodium deoxycholate, pH 7.4, with 1:500 EDTA-free protease inhibitor cocktail III (Calbiochem, 53914). The homogenate pellet was cleared by centrifugation at 17,000 RPM for 1 h at 4°C. Supernatants were aliquoted and stored at −80°C for later use.

### Western Blot Analysis

Protein expression in the cerebellum from the FRDAkd mouse was assessed as previously described (Lin et al., 2014). After tissue was lysed, protein concentration was determined using a BCA Protein Assay kit (Thermo Fisher Scientific, 23225). 20–30 micrograms of proteins were loaded into a 4–12% NuPAGE Gel SDS polyacrylamide gel. After electrophoresis, the proteins were transferred to a nitrocellulose membrane and incubated with anti-frataxin antibodies and anti-actin overnight at 4°C. The blots were later incubated in horseradish peroxidase (HRP)-conjugated secondary mouse or rabbit antibody (1:2,000 Cell Signaling Technology) using 3% skim milk diluted in Tween/Tris-buffered saline (TBS-T) for 1.5 h at room temperature and later washed with TBS-T. In addition, the blots were stripped with stripping buffer (2% SDS, 50 mM Tris, pH 6.8, and 100 mM β-mercaptoethanol) for 45 min at room temperature to remove the signals before probing for other proteins and later washed three times with TBS-T. The bands obtained were quantified using NIH ImageJ software^1^ and normalized to loading control-actin.

### Histological Analysis

For histological analysis, FRDAkd and control mice at 12-week of Dox-induction were anesthetized with isoflurane and cardiac perfused with 10 ml of PBS, followed by 20 ml of PBS containing 4% paraformaldehyde in accordance with protocols approved by the Children’s Hospital of Philadelphia’s Animal Care and Use Committee. Brains were excised and immersed overnight in 4% paraformaldehyde, washed in PBS, dehydrated, and embedded in paraffin. A series of brain and DRG sections (5 μm thick) were obtained using microtome at the Children’s Hospital of Philadelphia Pathology Core Facility (Lin et al., 2005).

Tissue pathology was assessed by performing immunohistochemical studies following the procedure previously described (Lin et al., 2014; Johnson et al., 2021). The paraffin-embedded brain and DRG sections were deparaffinized, rehydrated, and antigen-retrieved in antigen unmasking solution (Vector Laboratories) and then subject to the immunostaining procedure. For immunostaining, after blocking with 5% normal goat serum and 1% bovine serum albumin in combination with 0.3% (vol/vol) Triton X-100 in PBS at room temperature for 1 h, the coverslips or slides were incubated with primary antibodies (frataxin and ATP5A) at 4°C overnight and then secondary antibodies conjugated to Alexa fluor 488 (Invitrogen, A11029) or 594 (Invitrogen, A11036) at room temperature for 60–90 min. Following several washes with PBS, cells or slides were mounted with Vectashield with DAPI (Vector Laboratories, H-1200).

### Rotarod Test

Mice were acclimated to the testing room 30 min prior to the test. Apparatus (sidewalls, rod, and board) was wiped down with sani-cloths (or 70% EtOH) before each trial. Training occurred over 3 days and latency to fall was recorded each following week.

#### Day 1 – Habituation

A single trial was performed at a constant low speed (5 RPM) to allow for mice habituation to the equipment. Mice were placed on a Rotarod track running at 5 RPM for 100 s. Mice that fell off the rod more than 3 times during testing were excluded from further testing.

#### Days 2 and 3 – Training

Mice were placed onto stationary tracks to begin. The Rotarod was then run; set to accelerate from 5 to 40 RPM over 300 s. 3 trials were completed with an intertrial interval of > 15 min. Trials were terminated when a mouse fell off, made one complete revolution while hanging on the bar, or after 300 s.

#### Testing

After the training period, the mice were tested weekly to biweekly. Mice ran at 5 RPM for 100 s on the Rotarod to warm up before beginning the test. The Rotarod was set to accelerate from 5 to 40 RPM over 300 s, at a linear pace, in the recorded trials. Latency to fall was recorded upon trial termination, which was marked by a mouse either falling, making one full revolution around the Rotarod bar, or after completion of 300 s. An interval of > 15 min was maintained between each of the 3 trials.

### Statistical Analysis

Data were analyzed for statistical significance using Prism9 and STATA. Two-tailed, unpaired Student’s *t*-test, one-way ANOVA, Two-way ANOVA, two-way repeated-measures ANOVA, Tukey’s *post hoc*, and linear regressions were used to compare groups where appropriate.

## Results

### Doxycycline-Compounded Food Induces FRDA-Related Phenotypes in the FRDAkd Mouse Model

**FIGURE 1:**
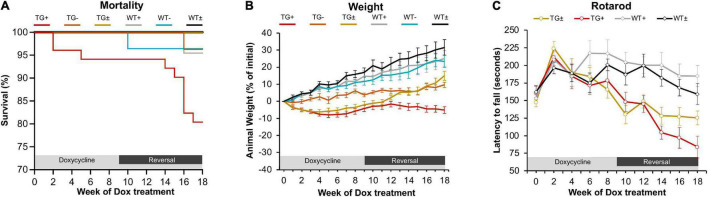
FRDAkd mice induced by doxycycline-compounded feed display FRDA-like phenotype **(A)** Survival rate of the FRDAkd mice after dox-feed induction. (TG + , *n* = 51; TG-, *n* = 27; TG ± , *n* = 13; WT + , *n* = 44; WT-, *n* = 28; WT ± , *n* = 14). **(B)** Animal weight throughout dox treatment and reversal in all groups (*n* = 21 TG + , *n* = 6 TG-, *n* = 13 TG ± , *n* = 17 WT + , *n* = 14 WT-, *n* = 14 WT ±). **(C)** Rotarod analysis throughout dox treatment and reversal in all groups TG ± , *n* = 8; TG + , *n* = 6; WT ± , *n* = 8; WT + , *n* = 8. Stats for panels C and D: Two-way repeated-measures ANOVA with Tukey’s *post hoc* (*p* values in Table 2). (TG + = transgenic with dox treatment for 18 weeks, TG- = transgenic no dox treatment, TG ± = transgenic with dox treatment for 9 weeks and reversal for subsequent 9 weeks, WT + = wild-type with dox treatment for 9 weeks, WT- = wild-type no dox treatment, WT ± = wild-type with dox treatment for 9 weeks and reversal for subsequent 9 weeks.) At week 0, all mice were between 2 and 5 months of age.

In previous studies, the FRDAkd mouse was induced by intraperitoneal dox injections and drinking dox water; in the present study, the dox-compounded feed was used, and this method generally reproduced the phenotype seen previously (Chandran et al., 2017). After 18 weeks of dox administration, transgenic mice (TG +) had a 19.6% mortality rate while the mortality rate of control groups was lower [transgenic without dox (TG-) and wild-type on dox (WT +] 3.7% and 4.5% respectively. Transgenic mice with dox removal at 9 weeks (TG ±) had no observable mortality (Figure 1A). One week after dox-treatment in 2–5-month-old mice, body weight progressively diminished in FRDAkd mice compared with control animals. On average TG + mice lost 5% body weight by 18 weeks of induction, while WT + gained 25.1%. This weight loss was reversible following dox removal within 4 weeks for initial dox induction, and TG ± body weight returned to near control mice weight (15.0% of baseline) (Figure 1B). Transgenic mice induced at 6 months or older of age were also evaluated for body weight change. TG + mice exhibited a 20.2% weight loss from their initial weight by 18 weeks of induction, while WT + gained 32% (Supplementary Figure 4). This suggests that the magnitude of weight loss was age-dependent, as mice induced at an age of 6 months or older lost approximately 4 times of initial body weight compared to mice induced at 2–5 months of age.

**TABLE 2:** *P* values for Two-way repeated-measures ANOVA with Tukey’s post hoc for animal weight and rotarod (Figures 1B,C).

	Weight *P-value*		
Week	TG + vs. WT +	TG ± vs. WT ±	TG + - vs. TG +
0	n.s.	n.s.	-
1	n.s.	n.s.	2.6e-06 ***
2	n.s.	n.s.	3.87e-11 ***
3	0.031*	n.s.	2.03e-11 ***
4	0.0114*	n.s.	5.39e-16 ***
5	0.0114*	n.s.	1.79e-13 ***
6	0.0073**	n.s.	1.31e-13 ***
7	0.0027**	0.0299*	6.72e-14 ***
8	0.007**	0.033*	1.4e-13 ***
9	0.0032**	0.0326*	5.56e-11 ***
10	0.0084**	0.0158*	6.98e-11 ***
11	0.0024**	0.025*	1.31e-09 ***
12	0.0084**	0.0222*	8.92e-09 ***
13	0.0059**	0.0365*	1.27e-08 ***
14	0.009**	0.0349*	1.63e-08 ***
15	0.0108*	0.0254*	6.99e-09 ***
16	0.008**	0.0317*	3.75e-10 ***
17	0.0053**	0.0311*	4.07e-10 ***
18	0.0025**	n.s.	3.5e-11 ***

	**Rotarod *P-*value**		
**Week**	**TG + vs. WT +**	**TG ± vs. WT ±**	**TG ± vs. TG +**

0	n.s.	n.s.	0.481
1	n.s.	0.0297*	-
2	n.s.	n.s.	0.0751
3	n.s.	n.s.	-
4	n.s.	n.s.	0.982
5	n.s.	n.s.	-
6	n.s.	n.s.	0.0111 *
7	n.s.	n.s.	-
8	n.s.	n.s.	0.00248 **
9	n.s.	n.s.	-
10	0.0187*	0.0472*	3.51e-06 ***
11	0.0325*	n.s.	-
12	-	-	0.00404 **
13	-	-	-
14	0.004***	0.0278**	0.000135 ***
15	0.0095**	n.s.	-
16	0.0035***	n.s.	3.16e-07 ***
17	0.0076**	n.s.	-
18	0.0002***	n.s.	2.43e-08 ***

Significance was set at *P < 0.05, **P < 0.01, and ***P < 0.0001.

FRDAkd mice displayed progressive motor dysfunction on the rotarod test. At 6 weeks of dox treatment, the latency of TG + mice to fall significantly decreased (172 ± 13 s) compared with WT (217 ± 18 s) and continued to decrease over the course of dox treatment. Reversal mice (TG ±) improved significantly (127 ± 12 s) compared to TG + mice (97 ± 15 s) mice by week 16 (7 weeks of reversal) and continued to improve through week 18. Dox removal did not affect WT + compared with WT ± mice (Figure 1C). Overall, both the change in weight and loss of motor skills began rapidly following induction, defining this as a model of sub-acute rather than chronic frataxin deficiency.

#### Tissue-Specific Frataxin Loss in Dox-Food Induced FRDAkd Mouse Model

**FIGURE 2:**
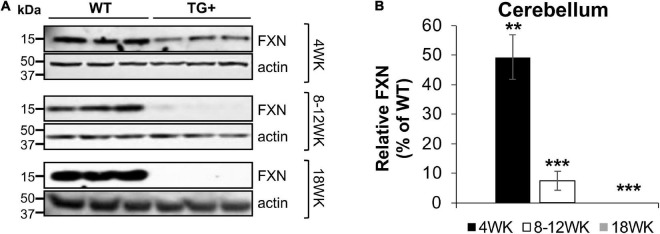
Dox treatment induces progressive frataxin deficiency in the cerebellum of FRDAkd mice. **(A)** Western blot analysis of frataxin (FXN) and internal loading control (actin) levels in the cerebellum of WT and TG + mice at 4 weeks, 8–12 weeks, and 18 weeks of dox treatment. **(B)** Quantification of frataxin levels normalized to internal control actin. The frataxin expression levels are reported as a percentage of the wild type at each time point set to 100 percent (Stats: WT, *n* = 3, TG +, *n* = 3, mean ± SEM, two-tailed, unpaired Student’s *t*-test, **P < 0.01, ***P < 0.001).

Frataxin levels decreased with dox treatment in a time-dependent manner in the central nervous system (CNS) and peripheral tissues. At 4 weeks of induction in the cerebellum, frataxin levels were reduced to 48.5% compared with control (WT + and WT-) tissues, with a reduction in the cerebellum at 8–12 (7.5% of control) and 18 weeks (0%) of induction. Peripheral frataxin levels declined more rapidly than levels in the CNS (Figures 2A,B and Supplementary Figures 1A,B) suggesting that tissue-specific effects from rapidly declining frataxin levels may appear in this model.

### Knockdown of Frataxin Increases Mitochondrial Fragmentation in the FRDAkd Mouse Cerebellum

**FIGURE 3:**
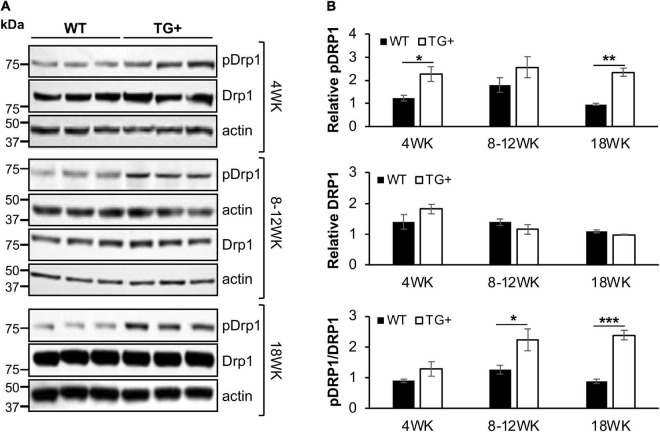
Mitochondrial fission protein Drp1 is progressively overactivated in the cerebellum of FRDAkd mice. **(A)** Western blot analysis of pDRP1 (S616), Drp1, and internal loading control (actin) levels in cerebellum of WT and TG + mice at 4 weeks, 8–12 weeks, and 18 weeks of dox treatment. **(B)** Quantification (normalized to actin) of pDRP1 (top panel), Drp1 (middle panel), and pDrp1/Drp1 ratio (bottom panel) normalized to actin (Stats: WT, *n* = 3, TG + , *n* = 3, mean ± SEM, two-tailed, unpaired Student’s *t*-test, *P < 0.05, **P < 0.01, ***P < 0.001).

**FIGURE 4:**
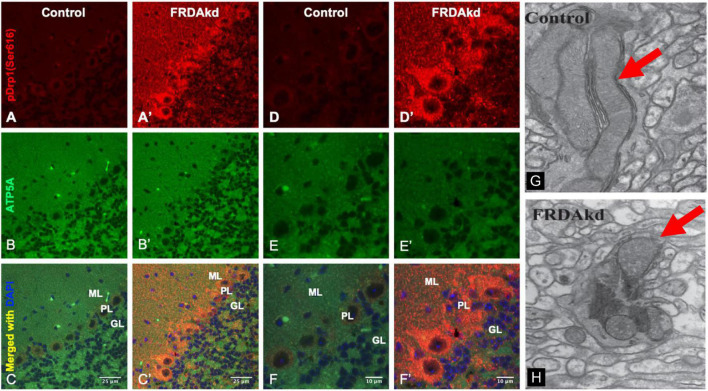
The cerebellum of FRDAkd mice display fragmented mitochondria and phospho-Drp1 (S616) increase localized to the cerebellar cortex and Purkinje neurons. Confocal images of phospho-Drp1 (S616) (red) and mitochondrial marker ATP5A (green) immunofluorescence and merged images with DAPI-stained nuclei (blue) in the cerebellar cortex and Purkinje neurons of FRDAkd **(A′–F′)** and age-matched control mice **(A–F)** at 8–12 weeks of dox treatment. Scale bars as indicated. Molecular layer (ML), Purkinje layer (PL), and granular layer (GL) are labeled. Electron microscopy images show fragmented mitochondria in FRDAkd mouse **(H)** cerebellar Purkinje neurons compared with age-matched control mice **(G)** at 18-weeks of dox treatment. Red arrows indicate mitochondria.

We previously reported that FRDA patient fibroblasts displayed high amounts of fragmented mitochondrial networks, accompanied by clustering of Dynamin-related protein 1 (Drp1) phosphorylated at its (Ser616) residue at the fragmentation sites (Johnson et al., 2021). Thus, to assess if frataxin knockdown manifests mechanistic similarities with *in vitro* experiments and leads to increases in pDrp1 in the FRDA cerebellum, we analyzed the cerebellum at different stages of induction in FRDAkd mice. Subacute frataxin knockdown with dox increased levels of this mitochondrial fission marker in the cerebellum of FRDAkd mice. At 4, 8–12, and 18 weeks of dox treatment, pDrp1 levels approximately doubled from baseline compared to wild-type while levels of total Drp1 did not change at any induction stage (Figures 3A,B). The ratio of pDrp1 to total Drp1 levels also increased, consistent with activation of Drp1 (Figure 3C). To establish the location in the cerebellum where such changes occurred, we performed immunohistochemistry; the increase in pDRP1 was localized most prominently to Purkinje cells with less prominent changes in granule cells and other areas (Figures 4A–F). Such biochemical abnormalities also appeared ultra-structurally. Transmission electron microscopy of the cerebellar cortex identified fragmented mitochondria in cerebellar Purkinje neurons of FRDAkd mouse cerebellum (Figures 4G,H). Thus, frataxin deficiency led to downstream structural changes in this region.

### Knockdown of Frataxin Leads to Cerebellar Synaptic and Neuronal Degeneration in the FRDAkd Mice

**FIGURE 5:**
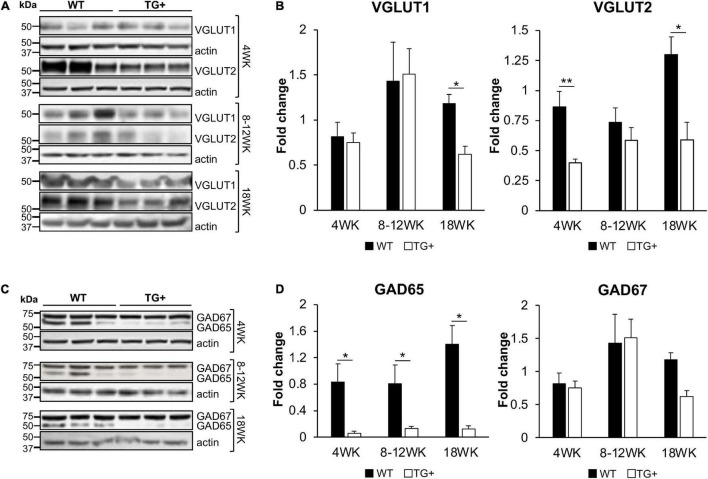
FRDAkd mice show progressive glutamatergic and GABAergic synaptic deficits in the cerebellum. Western blot and quantification of VGLUT1, VGLUT2 **(A,B)**, GAD65, and GAD67 **(C,D)** levels normalized to internal control actin in WT and TG + mice at 4 weeks, 8–12 weeks, and 18 weeks of dox treatment (Stats: WT, *n* = 3, TG +, *n* = 3, mean ± SEM, two-tailed, unpaired Student’s *t*-test, *P < 0.05, **P < 0.01).

**FIGURE 6:**
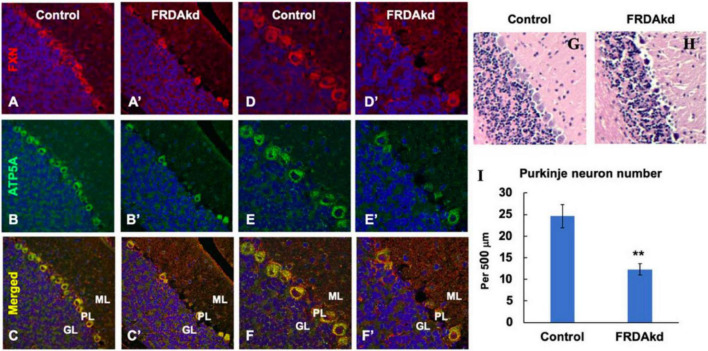
The number of ATP5A-positive-cerebellar Purkinje neurons is decreased in FRDAkd mice at 18 weeks of dox treatment. Confocal images of frataxin (FXN) (red) and mitochondrial marker ATP5A (green) fluorescence, and merged images with DAPI-stained nuclei (blue) in the cerebellum of FRDAkd **(A′–F′)** and control mice **(A–F)**. Hematoxylin-Eosin staining of cerebellar Purkinje neurons in FRDAkd **(H)** and control **(G)** mice. **(I)** Quantification of ATP5A-positive Purkinje neurons in FRDAkd and control mice at 18 weeks of dox treatment (Stats: *n* = 3 mice per group, 3 sections per mouse, mean ± SEM, student’s *t*-test, **P < 0.01).

**FIGURE 7:**
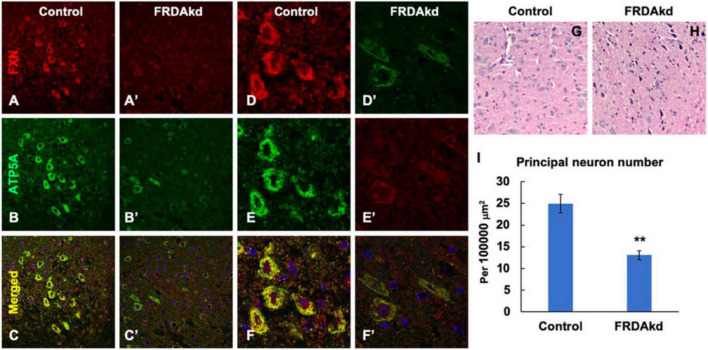
The number of frataxin- and ATP5A-positive-cerebellar dentate nucleus principal neurons is decreased in FRDAkd mice at 18 weeks of dox treatment. Confocal images of frataxin (FXN) (red) and mitochondrial marker ATP5A (green) fluorescence, and merged images with DAPI-stained nuclei (blue) in the cerebellum of FRDAkd **(A′–F′)** and control **(A–F)** mice. Hematoxylin-Eosin staining of cerebellar principal neurons FRDAkd **(H)** and control mice **(G)**. **(I)** Quantification of ATP5A-positive principal neurons in FRDAkd and control mice at 18 weeks of dox treatment (Stats: *n* = 3 mice per group, 3 sections per mouse, mean ± SEM, student’s *t*-test, **P < 0.01).

The synaptic integrity of the cerebellar cortex was also altered based on synaptic markers. Vesicular Glutamate Transporter 1 (VGLUT1), a marker of parallel fibers arising from cerebellar granule cells, remained unchanged compared to wild-type animals at early Dox induction (within 4 weeks) but decreased by and 18 weeks after induction (Figures 5A,B). In contrast, VGLUT2 and GAD65, markers of extrinsic cerebellar pathways and GABAergic interneurons respectively, decreased within 4 weeks of induction. GAD 67 levels, most prominent in Purkinje cells of the cerebellar cortex, remained unchanged (Figures 5C,D). Cerebellar neuronal death also appeared over time. By 18 weeks of induction, the number of *FXN*- and ATP5A-positive Purkinje cells is decreased to 60% of wild-type (Figures 6A–I), and the number of large *FXN*- and ATP5A-(a mitochondrial marker) positive principal neurons of the dentate nucleus decreased to 50% of wild-type (Figures 7A–I). Notably, the Purkinje or DN large principal neurons lacking frataxin exhibit loss of ATP5A-positive mitochondria in the soma. HE histological staining further confirmed degenerative Purkinje and DN large principal neurons in FRDAkd mouse cerebellum. This suggests that Dox-induced loss of frataxin leads to mitochondria loss and subsequent degeneration of cerebellar Purkinje neurons and DN large principal neurons at a late stage, implicating a significant role of frataxin in maintaining mitochondrial integrity and viability of Purkinje and DN large principal neurons.

### Failure of Frataxin Levels to Reverse With Dox Removal

**FIGURE 8:**
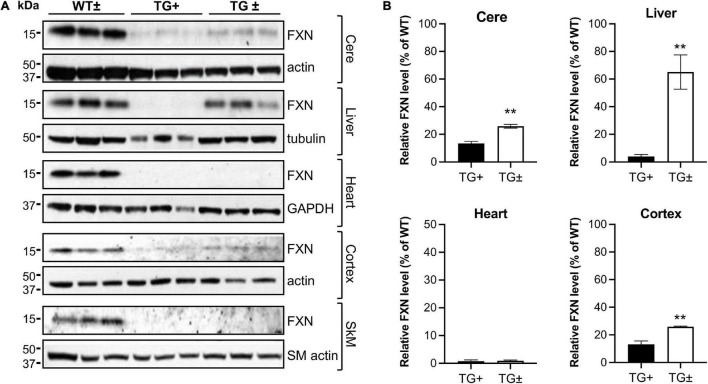
Dox reversal partially reverses frataxin deficiency in a tissue-specific manner in FRDAkd mice. **(A)** Western blot analysis in WT ±, TG + , and TG ± mice of frataxin and tissue-specific internal loading controls (actin, SM-actin, GAPDH, and tubulin) in the cerebellum (cere), liver, heart, cortex, and skeletal muscle (SkM) (*n* = 3 for WT ± , TG +, and TG ±). **(B)** Quantification of frataxin levels normalized to tissue-specific internal controls (actin, SM-actin, GAPDH, tubulin). Frataxin levels were reported as a percentage of the wild type set to 100 percent. Quantification of SkM was zero in both TG + and TG ± (not shown) (Stats: WT, *n* = 3, TG +, *n* = 3, TG ±, *n* = 3, mean ± SEM, two-tailed, unpaired Student’s *t*-test, **P < 0.01).

Upon removal of dox, the shRNA transgene in the FRDAkd mouse is repressed, potentially allowing recovery of frataxin levels and recovery of weight and motor coordination. However, after 9 weeks of dox administration, dox was removed, but frataxin expression levels in cerebellum, liver, heart, cortex, and skeletal muscle failed to return to normal levels (WT ±), only recovering partially in a tissue-specific manner. Frataxin levels in the TG ± liver recovered nearly completely compared to TG + mice (4.0 vs. 65.1% of WT ±, respectively), consistent with the original model (Chandran et al., 2017). Furthermore, frataxin levels also recovered to some degree after dox removal in TG ± mouse cerebellum and cortex (25.9% and 25.9% respectively), a modest recovery compared with TG + mice (13.1% and 13.4% respectively). Frataxin levels did not recover in TG + heart and skeletal muscle (Figures 8A,B).

### *In vivo* AAV8 Treatment Restores VGLUT1 and VGLUT2 Deficiency in the FRDAkd Mice

**FIGURE 9:**
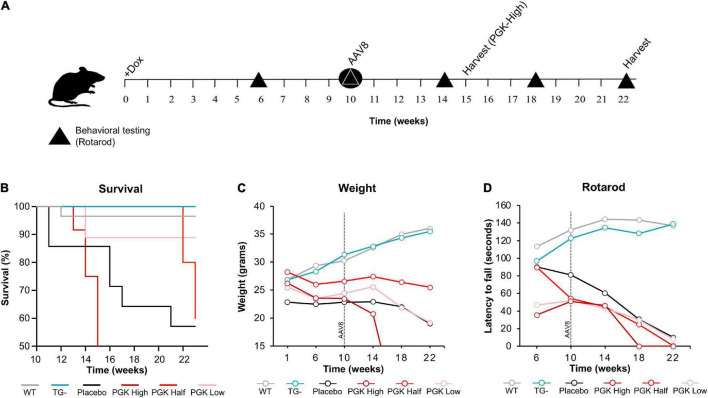
AAV8-m*FXN* gene therapy partially reverses reduced survival and weight loss in FRDAkd mice. **(A)** Experimental paradigm of gene therapy (AAV8-m*FXN*) treatment in 3-month-old FRDAkd mice. Doxycycline treatment started at week “0” and continued to week 22 (except PGK high which had early termination because they died after 15_weeks on dox/5 weeks after AAV8). All groups received AAV8 10 weeks post doxycycline treatment and rotarod testing was performed at weeks 6, 10, 14, 18, and 22 post dox treatment. **(B)** Survival rate, **(C)** animal weight and **(D)** rotarod analysis of AAV8-m*FXN* treated FRDAkd mice and controls. WT = wild-type, TG- = transgenic no dox treatment, PGK High = transgenic mice with dox treatment and 3.00E + 14 vg/kg AAV8, PGK Half = transgenic mice with dox treatment and 0.5 E + 14 vg/kg AAV8, PGK Low = transgenic mice with dox treatment and1.00E + 14 vg/kg dose AAV8, placebo = transgenic mice with dox treatment and placebo (Lactated Ringer’s Solution w/10% Poloxamer) [Stats: WT, *n* = 32, TG-, n = 13, TG- placebo *n* = 6, TG + PGK high, *n* = 9, TG + PGK low *n* = 9, Two-way repeated-measures ANOVA with Tukey’s *post hoc* for panels **(B,C)**, *P* values on Tables 3, 4].

**FIGURE 10:**
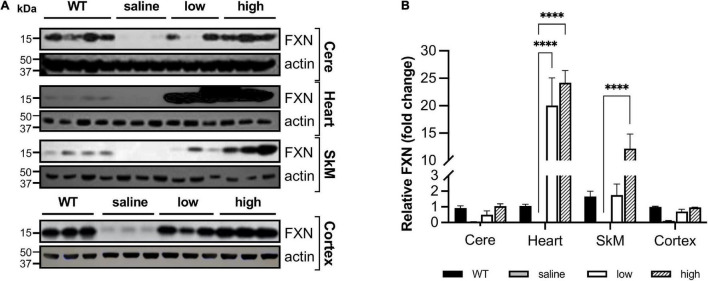
AAV8-m*FXN* gene therapy partially restores frataxin in peripheral and central nervous system tissues of FRDAkd mice **(A)** western blot analysis and **(B)** quantification of frataxin normalized to loading control (actin) in the cerebellum (Cere), heart, skeletal muscle (SkM), and cortex of wild-type (WT) mice, and transgenic (dox treated) mice treated with AAV8 low or high dose (Stats: WT = combined results for WT + and WT-, *n* = 4, TG + saline, *n* = 3, TG + low, *n* = 3, TG + high, *n* = 3, mean ± SEM, two-way ANOVA with Tukey’s *post hoc*, ns = not significant, ^****^P < 0.0001, stats displayed are comparing saline to low and high dose AAV8 treated mice).

Even though body weight, coordination, and mortality recovered almost immediately with dox removal (Figure 1), the failure of frataxin to recover rapidly confounds simple interpretation of the reversibility of the effects of frataxin deficiency. While dox administration to wild-type animals produced none of these features, the dox alone or the combination of dox administration and frataxin deficiency might mediate some of the effects. Consequently, we used gene therapy with AAV8-PGK frataxin *in vivo* in an attempt to rescue disease features in the FRDAkd mouse. This provides a reversal paradigm in which doxycycline remains present, but frataxin is restored, allowing assessment of the effects of frataxin alone. Intravenous administration of AAV8-PGK efficiently restored frataxin in diverse tissues of the FRDAkd mouse in a dose-dependent manner. Frataxin restoration was greatest in tissues of mice that received the highest dose of PGK vector (3.00E + 14 vg/kg) (Figures 9A–D) and progressively lower with lower doses (1.00E + 14 vg/kg and 0.5 E + 14 vg/kg). With high dose AAV8-PGK, frataxin levels were restored to at least those of wild-type animals in the brain, and more in other tissues. Among the tissues analyzed, the heart had the highest detectable frataxin, followed by skeletal muscle and cerebral cortex (Figures 10A,B).

**TABLE 3:** *P* values obtained from a two-way ANOVA on animal weight (Figure 9C).

	Week 1	Week 6	Week 10	Week 14	Week 18	Week 22
WT vs. Tg +	0.008086	4.15E-06	1.07E-06	2.23E-08	1.74E-08	7.95E-10
	**	***	***	***	***	***
WT vs. Tg-	0.989668	0.74143	0.448772	0.894038	0.785077	0.830688
	n.s.	n.s.	n.s.	n.s.	n.s.	n.s.
Tg + vs. Tg-	0.010103	0.000138	1.94E-05	1.19E-05	2.05E-05	4.63E-07
	*	***	***	***	***	***
Tg + vs. PGK low	0.071372	0.47456	0.248032	0.034477	0.903198	0.750925
	n.s.	n.s.	n.s.	n.s.	n.s.	n.s.
Tg + vs. PGK half	0.002171	0.017568	0.019533	0.00563	0.010417	3.05E-06
	**	*	*	*	*	***
Tg + vs. PGK high	0.015524	0.414668	0.657465	0.07893	-	-
	*	n.s.	n.s.	n.s.	n.s.	-

Significance was set at *P < 0.05, **P < 0.01, and ***P < 0.0001.

**TABLE 4:** *P* values obtained from a two-way ANOVA on animal behavior (Figure 9D).

	Week 6	Week 10	Week 14	Week 18	Week 22
WT and Tg +	0.03800095	2.53E-06	2.09E-11	2.13E-19	4.38E-20
	**	***	***	***	***
PGK half vs. Tg-	0.69854339	3.14E-06	1.63E-07	4.92E-10	1.92E-14
		***	***	***	***
PGK low vs. Tq-	4.32E-06	5.15E-09	9.89E-11	4.17E-12	2.08E-17
	***	***	***	***	***
Tg + vs. Tg-	0.61480229	0.000697121	9.44E-09	4.41E-15	1.00E-20
		**	***	***	***
PGK low vs. Tg +	0.00013403	0.005592562	0.08977842	0.899492814	0.46909738
	***	**	n.s.	n.s.	n.s.
PGK half vs. Tg +	0.98245877	0.051405024	0.23053219	0.399584418	0.00311152
	n.s.	n.s.	n.s.	n.s.	**
WT vs. Tg-	0.26381014	0.493564981	0.52770667	0.232862104	0.89209696
	n.s.	n.s.	n.s.	n.s.	n.s.
PGK high vs. Tg-	4.43E-08	3.33E-07	6.72E-13	–	–
	***	***	***	–	–
PGK high vs. Tg +	2.70E-06	0.009980835	0.16122373	–	–
	***	**	n.s.	–	–

Significance was set at *P < 0.05, **P < 0.01, and ***P < 0.0001.

Surprisingly, motor function, weight, and mortality changed little in Dox treated FRDAkd mice that received AAV8 treatment (Figures 9B–D). Over time, PGK-*FXN* high dose, developed the greatest weight loss, the largest latency to fall, and had the lowest survival rate. This suggests at least some toxic effect of this vector-induced highest expression of frataxin in these animals; this was confirmed by pathological analysis revealing histological evidence of toxicity in FRDAkd mice that was exacerbated by high dose PGK-*FXN* (Supplementary Figure 2). Lower doses of PGK-*FXN* had no clear deleterious or beneficial effect on survival rate, weight, motor function, or histological properties compared with FRDAkd-untreated, or placebo-treated.

**FIGURE 11:**
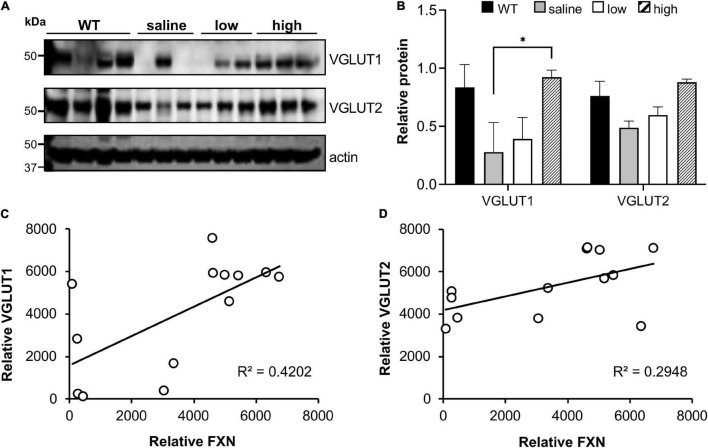
AAV8-mFXN gene therapy restores VGLUT1 expression in the cerebellum of FRDAkd mice. **(A)** western blot analysis and **(B)** quantification of VGLUT1 and VGLUT2 levels in AAV8-mFXN treated mice and controls (Stats: mean ± SEM, Two-way ANOVA with Tukey’s *post hoc*, *P < 0.05). Correlation plots comparing relative frataxin (FXN) levels with relative **(C)** VGLUT1 and **(D)** VGLUT2 levels in mice of all treatment groups (Stats: linear regression; frataxin vs. VGLUT1, *R*^2^ = 0.42, *P* = 0.016; linear regression; frataxin vs. VGLUT2, *R*^2^ = 0.29, *P* = 0.055; Spearman rank correlation; frataxin vs. VGLUT1, *R*_S_ = 0.015; Spearman rank correlation; frataxin vs. VGLUT2, *R* = 0.032).

We also investigated if frataxin restoration with AAV8 frataxin alone can reverse the biochemical features of FRDAkd mice. pDRP1 and DRP1 levels did not change with frataxin restoration in any tissue, or any vector administered (data not shown). In contrast, cerebellar synaptic markers (pooled VGLUT1 and VGLUT2) reversed to a modest degree (Figures 11A,B). VGLUT1 levels were restored substantially in animals that received a high dose of the PGK vector and in some animals that received a low dose of PGK. While VGLUT2 levels were restored to some degree with high dose PGK, GAD65 and GAD67 levels were not affected (data not included). In addition, the level of frataxin restoration significantly correlated with the level of VGLUT restoration (using data from all animals), suggesting that the exact level of frataxin in the cerebellum determined levels of these synaptic markers (Figures 11C,D).

## Discussion

The FRDAkd mouse model, induced through a doxycycline-compounded feeding method, results in frataxin deficiency, allowing it to model some of the behavioral and particularly cerebellar deficits noted in human FRDA. The present model produces profound frataxin deficiency associated with a significant behavioral phenotype similar to results shown in previous studies (Chandran et al., 2017). However, the degree of weight loss seen here is less than that seen with the dox injection/drinking method, particularly earlier on in the induction, when initial dosing of dox led to a sharp decrease in frataxin levels (Chandran et al., 2017). Overall, these results indicate that not only the level of frataxin deficiency but also the rate at which it is lost, are important contributors to the resulting phenotype in FRDAkd mice. The FRDAkd mouse should provide the most insight into the degenerative aspects of FRDA without capturing developmental components. Thus, the phenotypic elements and in particular, the rate of their progression in FRDAkd mice may differ from human FRDA, in which frataxin deficiency is chronic and minimally progressive. Consequently, frataxin deficiency in FRDAkd mice will inherently produce distinct phenotypic features from those of human disease. For example, histological assessment of FRDAkd mice demonstrated loss of a variety of specific cells, many, but not all, of which are also affected in FRDA (Cawthorne et al., 2007; Redelsperger et al., 2016; Thomas-Black et al., 2019). Thus, the present approach creates a subacute frataxin model useful for further study of frataxin deficiency-dependent neurodegeneration and with specific caveats.

The challenge of re-creating all the features displayed by FRDA patients in mouse models has been previously reported (Chandran et al., 2017). In the present report, neuropathological analysis of FRDAkd mice identifies loss of cerebellar Purkinje neurons and large dentate nucleus principal neurons as well as large DRG sensory neurons, but only the latter two mimic neuropathology from patients with FRDA (Koeppen et al., 2011; Harding et al., 2020; Supplementary Figure 3). However, the Purkinje cell loss does match results in neuron-specific frataxin knockout mice and parvalbumin-based conditional frataxin-knockout mice (Simon et al., 2004; Piguet et al., 2018). FRDA KIKO mice, which contain a knockout frataxin allele and GAA repeat sequences, have 25–30% residual frataxin compared to WT levels and a mild behavioral phenotype (Lin et al., 2017, 2018), and exhibit less severe features than patients with FRDA. In the KIKO mouse model, Purkinje cells remain intact but have diminished levels of parvalbumin (Lin et al., 2017, 2018). This more closely matches human FRDA, as the Purkinje cells are altered neuropathologically in patients. The present model suggests that changes in Purkinje cells could exist in FRDA patients but are perhaps not associated with frank neuropathological features or are obscured by other disease features such as loss of large dentate nucleus neurons. The absence of developmental aspects in the FRDAkd model suggests that effects on Purkinje cells are neurodegenerative rather than neurodevelopmental in origin.

Thus, the overall pattern of structural changes in the cerebellum of FRDAkd mice is only partially reminiscent of human FRDA in other ways beyond effects on Purkinje cells. While the loss of Purkinje cells in FRDAkd animals is still unexpected, the preservation of GAD67 levels suggests that the remaining Purkinje cells have increased levels of GAD (Figures 5C,D, 6A–I). Reactive changes consistent with regeneration have been noted in the cerebellar cortex of FRDA patients, suggestive of repair of Purkinje cells (Koeppen et al., 2011; Kemp et al., 2016; Harding et al., 2020). The loss of large dentate nucleus neurons found here is a clinical and pathological hallmark of FRDA that is also found in KIKO mice (Koeppen et al., 2011; Kemp et al., 2016; Lin et al., 2017, 2018; Harding et al., 2020). GAD65 loss from GABAergic interneurons is also found in KIKO mice, suggesting a uniform disruption in these synapses in multiple FRDA models (Lin et al., 2017, 2018). However, the immediate loss of VGLUT2 from extrinsic inputs (climbing fibers) in FRDAkd mice was not found in KIKO mice (Figures 5A,B; Lin et al., 2017). This pathway is also not affected to a large degree in patients with FRDA. Both GAD65 and VGLUT2 increase in KIKO mice, consistent with an early developmental component to cerebellar disruption (Lin et al., 2017). This developmental compensation matches present views on early involvement of developmental events in FRDA (Koeppen et al., 2011; Marty et al., 2019; Harding et al., 2020; Naeije et al., 2020, 2021). Thus, one possibility for the divergent results from FRDAkd mice is that the relatively severe frataxin deficiency or the accelerated rate of decline leads to a greater acute level of neuropathological features in Purkinje cells and climbing fibers than typically noted in FRDA patients or other mouse models (Koeppen et al., 2011; Harding et al., 2020). In addition, the late induction of frataxin deficiency in the FRDAkd mouse does not allow for developmental effects and may target pathways more vulnerable later in life. Consequently, cerebellar pathology of FRDAkd mice parallels only selected parts of FRDA.

While the FRDAkd model has certain advantages over repeat-expansion-based chronic models (YG8R, YG22R, and KIKO), the present study demonstrates that it also possesses many disadvantages, perhaps reflecting the sub-acute nature of this model (Al-Mahdawi et al., 2004, 2006; Lin et al., 2017, 2018). Frataxin was almost, if not completely, lost in all tissues in the mouse after 9 weeks of Dox induction (Figures 2A,B and Supplementary Figure 1). Due to this dramatic loss of frataxin, the disease recapitulated was one of sub-acute frataxin knockout rather than chronic frataxin deficiency seen in FRDA. As a result, the phenotypes observed in this model may be extreme. Photoreceptor death and retinal pigment epithelial (RPE) layer degeneration occur in the FRDAkd mouse model, while in humans only the retinal ganglion cells exhibit increased cell death (Noval et al., 2012; Seyer et al., 2013; Chandran et al., 2017; Hamedani et al., 2018; Thomas-Black et al., 2019). However, this observation may also reflect species differences since RPE thinning also occurs in YG8R and YG22R, and the death of photoreceptors has also been observed in YG22R mice (Al-Mahdawi et al., 2004, 2006).

In general, mortality, age-dependent body weight loss, and severe motor coordination deficits are comparable between the previous and present models of dox induction, but there are some key differences. While similar to previously reported, the early mortality (within the first month after induction) was lower using feed-induced animals than in reports with intraperitoneal injection/drinking water induced frataxin reduction (19.6% compared to ∼50% mortality rate, respectively) (Figure 1A; Chandran et al., 2017). This could conceivably reflect acute frataxin reduction from injection. Mouse manipulation can cause handling-associated stress; circumventing such stress on the animals provides a more ethical and reproducible experimental paradigm (Cawthorne et al., 2007; Redelsperger et al., 2016). Overall, both the change in weight and loss of motor skills began rapidly following induction, defining this as a model of sub-acute rather than chronic effects of frataxin reduction.

In the present model, the rate of decline in frataxin levels varies across tissues analyzed after Dox induction of the shRNA transgene (Supplementary Figure 1). The reason for these anomalies is unclear. It may reflect uneven dox distribution and partitioning, differing rates of frataxin turnover, or numerous other factors including tissue-specific mechanisms for controlling frataxin expression. In addition, because of the liver’s propensity to regenerate in the presence of complete frataxin deficiency, this tissue might exhibit some frataxin recovery by selecting the few daughter cells in which the shRNA transgene is dropped over multiple cell divisions (Martelli et al., 2012). Still, despite the slow onset of frataxin deficiency in the CNS, frataxin levels parallel the onset of coordination deficits observed in these mice.

Reversal of frataxin knockdown with dox removal shows an incomplete, tissue-specific frataxin recovery. In the cerebellum and cortex, there is a slow, significant rescue of frataxin levels, yet they remain diminished compared to WT (Figures 8A,B). However, little frataxin recovery is observed in the heart and skeletal muscle, but frataxin does recover in the liver. Surprisingly, although frataxin was restored incompletely and inconsistently across some tissues and not at all in other tissues, behavioral and andromorphic deficits improved following removal of dox at a much faster rate than CNS frataxin recovered (Figures 1B,C). In the previous work on this model, frataxin restoration in the liver was incomplete (only 40%) at 8 weeks after removal of dox (Chandran et al., 2017). Although the exact reason for incomplete frataxin restoration after discontinuation of dox is unclear, there are a variety of possibilities. It could reflect persistent dox storage in certain tissues (Cawthorne et al., 2007; Redelsperger et al., 2016). Chronic dox exposure also might substantially alter cellular or mitochondrial metabolism when given in combination with frataxin deficiency. Dox inhibits mitochondrial biogenesis and mitochondrial protein synthesis and can cause mitochondrial damage (Anders et al., 2012; Ahler et al., 2013; Jasoliya et al., 2017; Luger et al., 2018; Scatena et al., 2018). In frataxin deficient mitochondria, dox could exacerbate damage and stress in certain cell types and account for the slow recovery of frataxin levels. In addition, phenotypic aspects improve following removal of dox, but not with gene therapy that rapidly corrects frataxin levels in the presence of dox (Figures 1, 9). This suggests that the behavioral and anthropomorphic abnormalities of this model do not reflect frataxin deficiency alone and involve complex effects of the temporal course of restoration and concomitant toxicity of doxycycline.

Overall, restoration of frataxin alone with AAV8-m*FXN* failed to improve most of the behavioral, metabolic, histological features of FRDAkd mice, suggesting that both frataxin deficiency and doxycycline administration contribute to these features of the FRDAkd model. Moreover, at high doses of AAV8-m*FXN* gene therapy that led to > 20-fold increase of frataxin in the heart, there was a suggestion of toxicity in this mouse model (Figures 9, 10). Consistent with the idea that high levels of frataxin can be deleterious to animals and cells (Lim et al., 2007; Perdomini et al., 2014; Belbellaa et al., 2019, 2020). We also investigated if frataxin restoration alone can reverse the biochemical features of FRDAkd mice. pDRP and DRP levels did not change with frataxin restoration in any tissue, or any vector administered (data not shown). In contrast, cerebellar synaptic markers reversed to a modest degree. VGLUT1 was restored substantially in all the animals that received a high dose of the AAV8-m*FXN* and in some animals that received a low dose (Figure 11). While VGLUT2 was restored to some degree in all the animals, GAD 65/67 was not affected (data not shown). In addition, (when looking at animals from all groups together) the level of frataxin restoration positively correlated with the level of VGLUT restoration (Figure 11). Taken together these results demonstrate that AAV8-m*FXN* restores frataxin protein expression and some cerebellar synaptic markers. Still, the major controller of phenotype appears to reflect the combination of doxycycline administration and frataxin deficiency.

## Data Availability Statement

The raw data supporting the conclusions of this article will be made available by the authors, without undue reservation upon request.

## Ethics Statement

The animal study was reviewed and approved by Animal Care and Use committee, Children’s Hospital of Philadelphia.

## Author Contributions

EM-A, NW, and SH completed the experiments. HL completed experiments on Figures 6, 7. EM-A and DL drafted the initial manuscript. LR provided statistical expertise, data interpretation, and figure preparation. All authors helped with the experiments and provided critique and feedback on the initial and subsequent versions of the manuscript.

## Conflict of Interest

FM, CF, and JC were employees of Audentes/Astellas at the time the work was performed. The remaining authors declare that the research was conducted in the absence of any commercial or financial relationships that could be construed as a potential conflict of interest.

## Publisher’s Note

All claims expressed in this article are solely those of the authors and do not necessarily represent those of their affiliated organizations, or those of the publisher, the editors and the reviewers. Any product that may be evaluated in this article, or claim that may be made by its manufacturer, is not guaranteed or endorsed by the publisher.

## Footnotes

1
https://imagej.nih.gov/ij/

## Supplementary Material

Supplementary Figure 1 Time-course of frataxin knockdown in CNS and peripheral tissue of FRDAkd mice at 4, 8–12, and 18 weeks of induction. **(A)** Western blot analysis of frataxin and internal loading (Actin) levels in cerebellum, DRG, spinal cord (SC), and cortex. **(B)** Quantification analysis of frataxin levels normalized to internal control actin. **(C)** Western blot analysis of frataxin and internal loading control (SM-Actin, GAPDH, and Tubulin respectively) levels in heart, skeletal muscle, and liver. **(D)** Quantification analysis of frataxin levels normalized to internal control (SM-Actin, GAPDH, Tubulin respectively). The frataxin expression levels are reported as a percentage to mean values of wild-type mice as 100 percent (**P* < 0.05, ^**^*P* < 0.01, ^***^*P* < 0.001, #*P* < 0.05, ##*P* < 0.001, two-tailed, unpaired Student’s *t*-test, error bars represent mean ± SEM for each panel. WT, *n* = 3, TG+, *n* = 3).

Supplementary Figure 2 Pathological analysis revealing histological evidence of toxicity in FRDAkd mice that was exacerbated by high dose PGK-*FXN.*

Supplementary Figure 3 Neuronal loss in the cerebellar cortex, dentate nucleus, and DRG of Dox-compounded feed FRDAkd mice at 12-week of Dox-induction. Hematoxylin-Eosin staining shows loss of cerebellar Purkinje neurons **(B)**, large DN principal neurons **(D)**, and large DRG sensory neurons **(F)** compared with those neurons in control mice **(A,C,E)**. Scale bars as indicated (25 μm).

Supplementary Figure 4 Older FRDAkd mice induced by dox-compounded feed display severe body weight loss. TG+ mice older than 6 months of age at Dox-induction show a higher percentage of weight loss of over 20% at 18 weeks compared with those at 2–5 months of age. (TG+, *n* = 17; TG-, *n* = 17; WT+, *n* = 10; WT-, *n* = 10). **P* < 0.05,***P* < 0.01,****P* < 0.001, ns not significant. One way ANOVA test followed by *t*-test.
