# Supplementary figures and images for: Cerebellar Pathology in an Inducible Mouse Model of Friedreich Ataxia
Source: Front Neurosci. 2022 Mar 24;16:819569. doi: 10.3389/fnins.2022.819569 (PMC8987918; doi:10.3389/fnins.2022.819569)

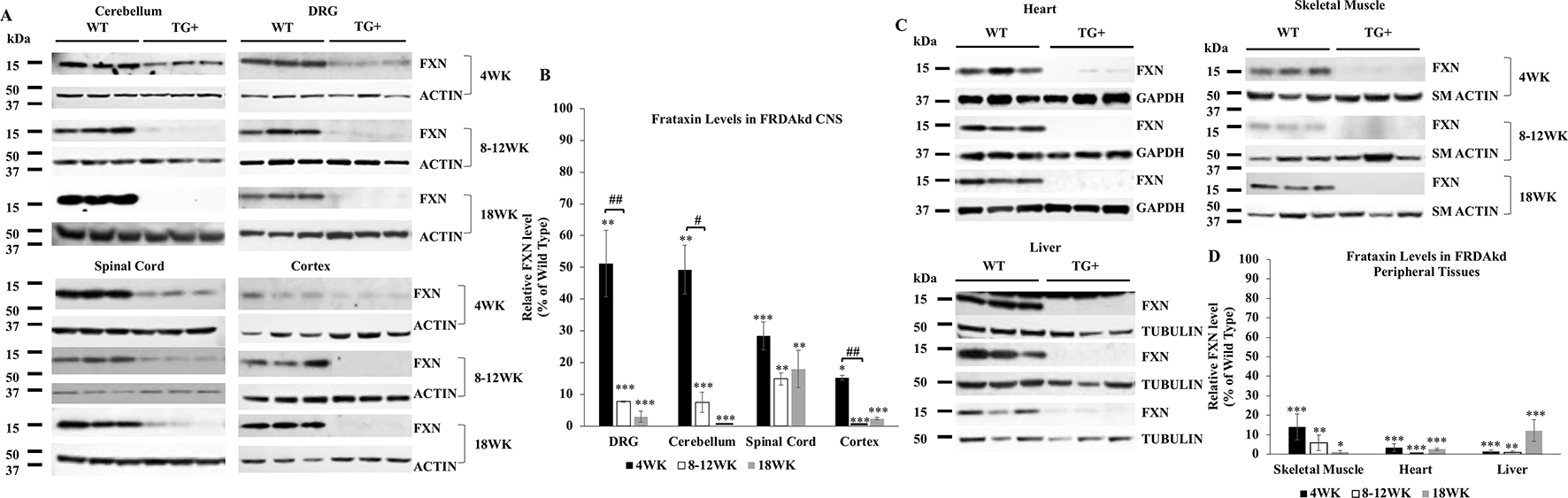

Supplement: Supplementary Figure 1 — Time-course of frataxin knockdown in CNS and peripheral tissue of FRDAkd mice at 4, 8–12, and 18 weeks of induction. (A) Western blot analysis of frataxin and internal loading (Actin) levels in cerebellum, DRG, spinal cord (SC), and cortex. (B) Quantification analysis of frataxin levels normalized to internal control actin. (C) Western blot analysis of frataxin and internal loading control (SM-Actin, GAPDH, and Tubulin respectively) levels in heart, skeletal muscle, and liver. (D) Quantification analysis of frataxin levels normalized to internal control (SM-Actin, GAPDH, Tubulin respectively). The frataxin expression levels are reported as a percentage to mean values of wild-type mice as 100 percent (*P < 0.05, **P < 0.01, ***P < 0.001, #P < 0.05, ##P < 0.001, two-tailed, unpaired Student’s t-test, error bars represent mean ± SEM for each panel. WT, n = 3, TG+, n = 3). [file Image_1.TIF]

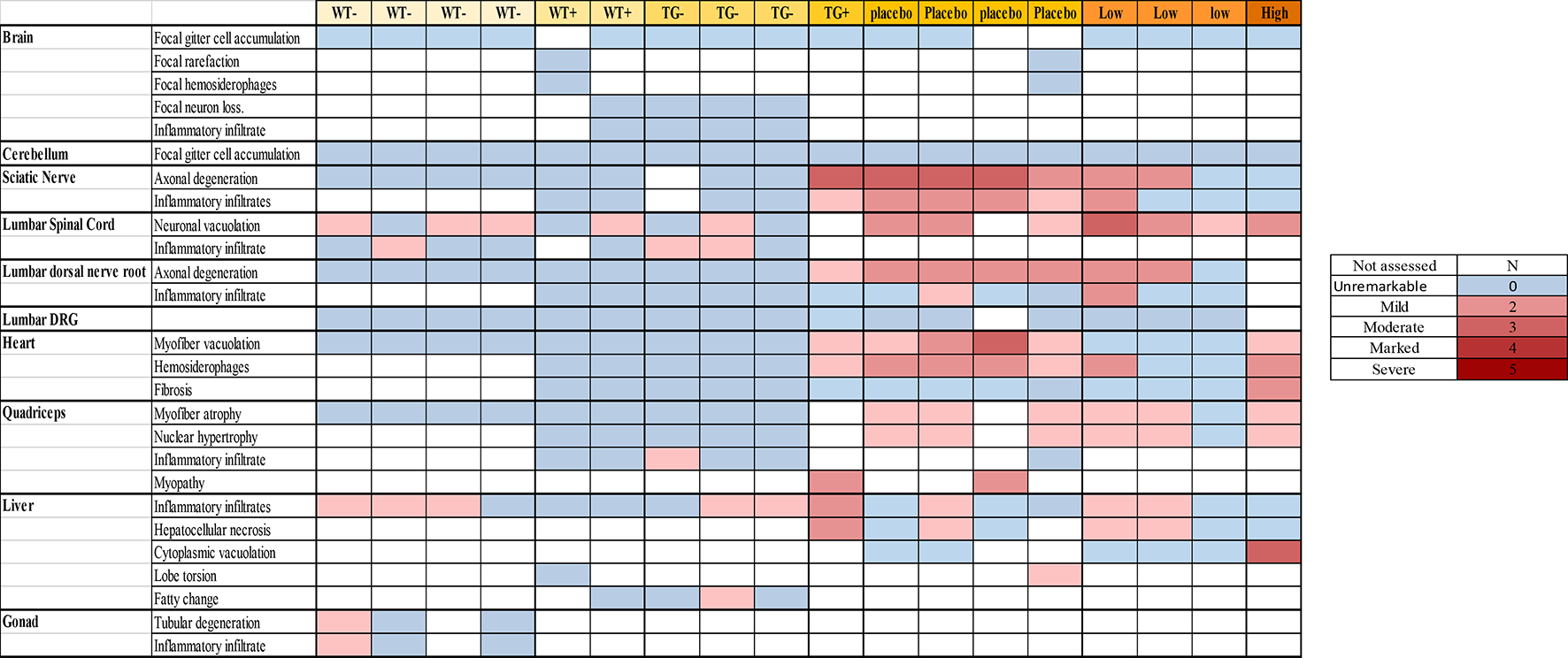

Supplement: Supplementary Figure 2 — Pathological analysis revealing histological evidence of toxicity in FRDAkd mice that was exacerbated by high dose PGK-FXN. [file Image_2.TIF]

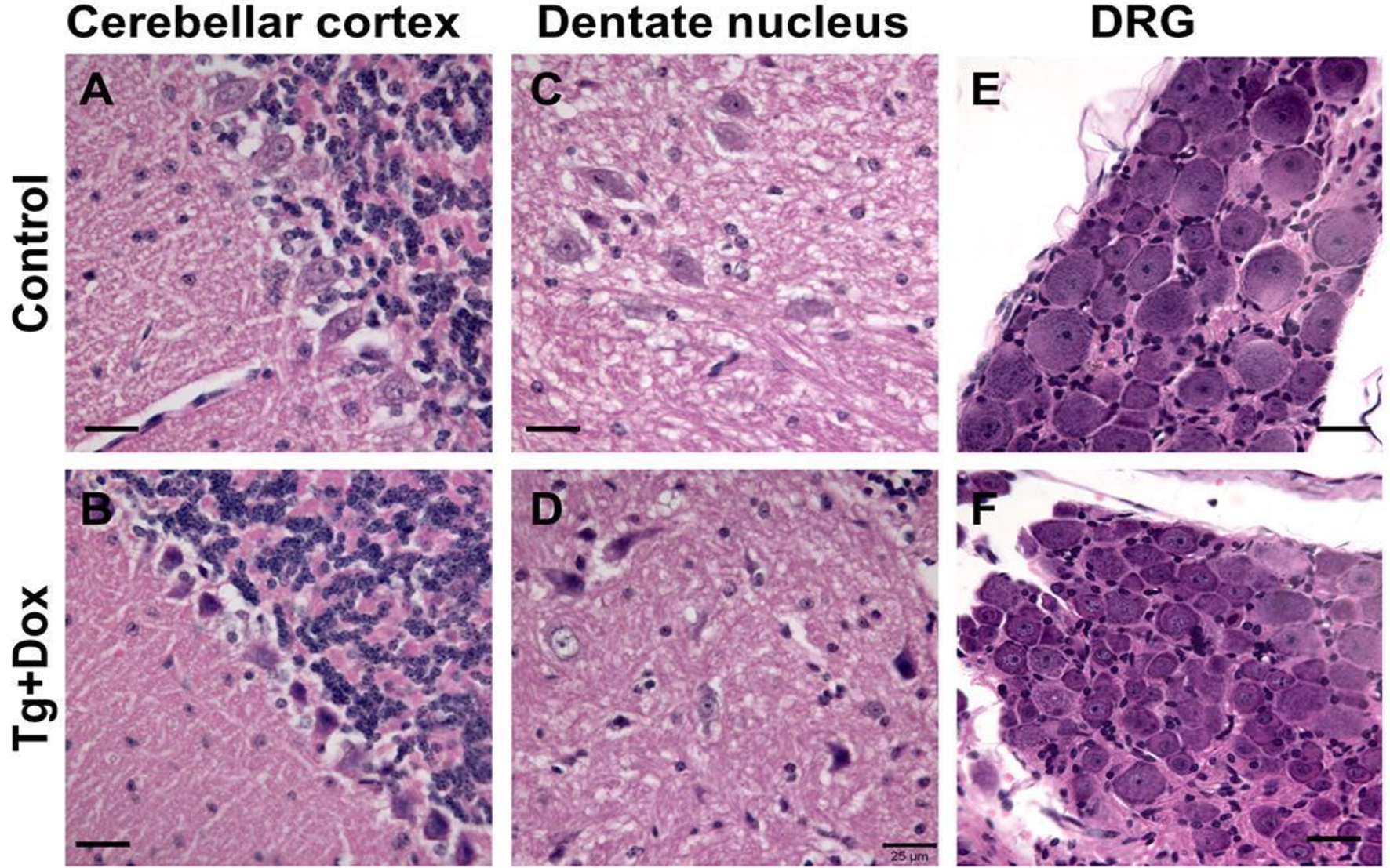

Supplement: Supplementary Figure 3 — Neuronal loss in the cerebellar cortex, dentate nucleus, and DRG of Dox-compounded feed FRDAkd mice at 12-week of Dox-induction. Hematoxylin-Eosin staining shows loss of cerebellar Purkinje neurons (B), large DN principal neurons (D), and large DRG sensory neurons (F) compared with those neurons in control mice (A,C,E). Scale bars as indicated (25 μm). [file Image_3.TIF]

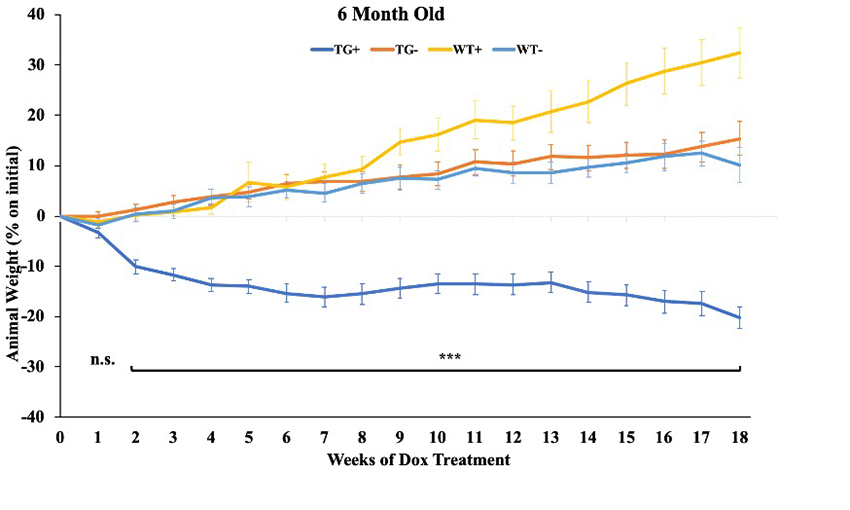

Supplement: Supplementary Figure 4 — Older FRDAkd mice induced by dox-compounded feed display severe body weight loss. TG+ mice older than 6 months of age at Dox-induction show a higher percentage of weight loss of over 20% at 18 weeks compared with those at 2–5 months of age. (TG+, n = 17; TG-, n = 17; WT+, n = 10; WT-, n = 10). *P < 0.05,**P < 0.01,***P < 0.001, ns not significant. One way ANOVA test followed by t-test. [file Image_4.TIF]
